# In Vitro Investigation of Novel Peptide Hydrogels for Enamel Remineralization

**DOI:** 10.3390/gels11010011

**Published:** 2024-12-27

**Authors:** Codruta Sarosi, Alexandrina Muntean, Stanca Cuc, Ioan Petean, Sonia Balint, Marioara Moldovan, Aurel George Mohan

**Affiliations:** 1 Department of Polymer Composites, Institute of Chemistry Raluca Ripan, Babes Bolyai University, 30 Fantanele Street, 400294 Cluj-Napoca, Romania; liana.sarosi@ubbcluj.ro (C.S.); stanca.boboia@ubbcluj.ro (S.C.); sonia.balint@ubbcluj.ro (S.B.); marioara.moldovan@ubbcluj.ro (M.M.); 2 Department of Paediatric Dentistry, Faculty of Dental Medicine, Iuliu Hatieganu University of Medicine and Pharmacy, 31 A. Iancu Street, 400083 Cluj-Napoca, Romania; 3 Faculty of Chemistry and Chemical Engineering, Babes-Bolyai University, 11 Arany Janos Street, 400028 Cluj-Napoca, Romania; ioan.petean@ubbcluj.ro; 4 Faculty of Medicine and Pharmacy, University of Oradea, P-ta 1 Decembrie 10, 410087 Oradea, Romania; mohanaurel@yahoo.com

**Keywords:** hydrogel, peptide, AFM, SEM, remineralization

## Abstract

This study investigates the microstructure of dental enamel following demineralization and re-mineralization processes, using DIAGNOdent scores and images obtained via scanning electron microscopy (SEM), atomic force microscopy (AFM), and microhardness (Vickers). The research evaluates the effects of two experimental hydrogels, Anti-Amelogenin isoform X (ABT260, S1) and Anti-Kallikrein L1 (K3014, S2), applied to demineralized enamel surfaces over periods of 14 and 21 days. The study involved 60 extracted teeth, free from cavities or other lesions, divided into four groups: a positive group (+), a negative group (−) and groups S1 and S2. The last three groups underwent demineralization with 37% phosphoric acid for 20 min. The negative group (−) was without remineralization treatment. The DIAGNOdent scores indicate that the S1 group treated with Anti-Amelogenin is more effective in remineralizing the enamel surface compared to the S2 group treated with Anti-Kallikrein. These findings were corroborated by SEM and AFM images, which revealed elongated hydroxyapatite (HAP) nanoparticles integrated into the demineralized structures. Demineralization reduced enamel microhardness to about 1/3 of a healthy one. Both tested hydrogels restored enamel hardness, with S1 being more effective than S2. Both peptides facilitated the interaction between the newly added minerals and residual protein binders on the enamel surface, thereby contributing to effective enamel restoration.

## 1. Introduction

Human enamel has a hierarchical structure composed of hydroxyapatite prisms packed together into a compact and dense structure. Each prism is about 5 µm in diameter and contains a dense mass of nanostructural clusters around 40–60 nm in size. These nanostructural clusters are formed by hydroxyapatite nanoparticles of about 20 nm, which are embedded together by a protein matrix [1,2].

Tooth remineralization is a natural process that restores minerals lost during acid-induced demineralization. Saliva neutralizes acids, raises oral pH, and supplies essential ions like calcium (Ca^2+^), phosphate (PO_4_^3−^), and fluoride (F^−^). These ions integrate into the demineralized enamel, forming fluorapatite, a more acid-resistant mineral. This strengthens the enamel, repairs early lesions, and creates a dense, protective layer to prevent further mineral loss [3,4,5].

Dental caries, one of the most prevalent diseases worldwide, affects approximately 35% of people, despite a decline in many regions. This pathophysiological condition arises from an imbalance in the demineralization–remineralization equilibrium within dental biofilms, leading to mineral loss and cavity formation. A primary driver of this process is the activity of over 750 bacterial species present in dental plaques, with Streptococcus species being the most predominant. These bacteria produce acids that demineralize tooth tissues, posing a significant challenge to oral health management [5,6,7].

To combat dental caries, various treatments have been developed, utilizing antibacterial agents, remineralization additives, and bioactive materials. For instance, calcium silicate-based products are widely used for their antibacterial, antioxidant, and remineralization properties, while root canal treatments employ antibacterial solutions to eradicate resistant bacteria [8,9]. However, some of these materials exhibit high cytotoxicity, limiting their widespread application. Despite these advancements, untreated cavities remain a significant global health issue, underscoring the need for innovative solutions that address the root causes of caries while minimizing adverse effects [10,11].

This growing challenge presents an opportunity to explore multifunctional biomaterials that integrate antibacterial, remineralization, and biofilm-disrupting properties. By leveraging advances in nanotechnology and biocompatible materials, it is possible to create targeted, effective, and safe solutions to manage dental plaques and caries more comprehensively [12,13].

Biomimetic remineralization, utilizing dentin phosphoprotein, shows promise as an anti-caries agent. By binding specifically to hydroxyapatite, it attracts free ions from artificial saliva, enhancing the surface properties of acid-eroded enamel and promoting remineralization. Remineralization depends on the presence of the same ions to restore the missing or damaged rods, ideally with fluoride acting as a catalyst [14,15].

The initial stages of demineralization induce morphological changes in enamel that are undetectable to the human eye. For an accurate and objective enamel surface characterization, visual methods are complemented with instrumental ones like the DIAGNOdent device, an optical laser-based fluorescence instrument that detects demineralized carious lesions by analyzing the intensity of reflected fluorescence from tooth structure. DIAGNOdent is used by practitioners not only to detect enamel condition but also to motivate the patient to follow an accurate oral hygiene regimen [16,17].

Amelogenin-derived peptides are short protein fragments derived from amelogenin, a key protein in enamel development. These peptides play roles in enamel mineralization and regeneration and are studied for dental repair and biomimetic applications. Gungormus et al. [18] developed a 22-amino-acid synthetic peptide, ADP-5, which promotes cementum-like hydroxyapatite formation on demineralized root dentin. Amin et al. [19] highlighted the potential of synthetic peptides in periodontal repair [20,21]. Anti-Kallikrein L1 peptides inhibit Kallikrein-related peptidase 1 (KLK1), an enzyme involved in inflammation, blood pressure regulation, and tissue remodeling. These peptides are studied for therapeutic applications in conditions like inflammatory diseases [22].

Peptide mediation of the organic binder within demineralized enamel is the novel pathway for hydrogel development, ensuring a better-quality treatment that is less invasive and does not cause pain. In consequence, this study aims to assess the impact of different peptides, Anti-Amelogenin and Anti-Kallikrein, incorporated into our novel hydrogels on the remineralization of demineralized enamel surfaces. The amino acid sequence provides more insight regarding Anti-Amelogenin and Anti-Kallikrein action during remineralization and requires further detailed investigation. The current study is more oriented on the investigation of the remineralization process results. This includes assessing demineralization levels using DIAGNOdent, analyzing surface properties with atomic force microscopy (AFM) and scanning electron microscopy (SEM), and measuring changes in surface roughness and hardness to determine the efficacy of the treatments. The null hypothesis comprises two parts: The first states that Anti-Amelogenin and Anti-Kallikrein have no effect on enamel surface parameters such as roughness and micro-hardness. The second part states that there is no difference between the action of Anti-Amelogenin regarding Anti-Kallikrein.

## 2. Results and Discussion

### 2.1. Enamel Surface Quality Assessment

**Figure 1:**
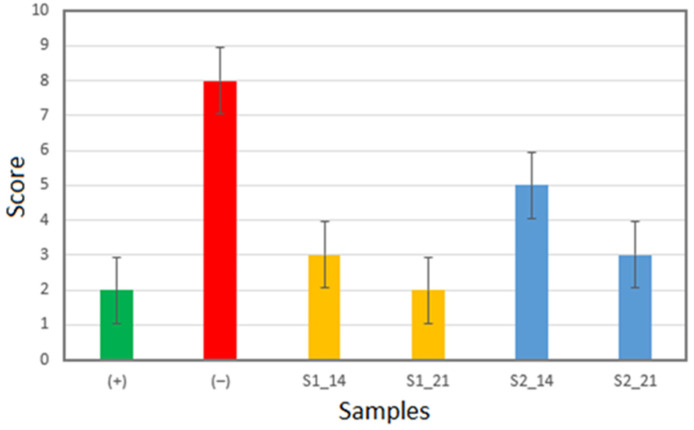
DIAGNOdent evaluation of the enamel surface.

Figure 1 presents the evaluation of the enamel surface after demineralization and remineralization at 14 and 21 days, using experimental hydrogels assessed with a DIAGNOdent pen. Score 1, with values between 0 and 4, represents healthy tooth structure, while score 2, with values between 5 and 10, represents outer half enamel caries.

DIAGNOdent measurements are sensitive to the condition of the enamel surface, and the scores reflect these aspects. A low value (0–4) indicates a healthy tooth structure. Increased scores indicate surface corrugation that is related to de-mineralization features. Thus, all values increased after demineralization, resulting in a score of 1 between 5 and 10, which indicates early surface caries involving half of the enamel thickness.

Healthy enamel, the positive control (+), has a score about of 2, and the demineralized enamel, the negative control (−), has score of 8. DIAGNOdent shows that Anti-Amelogenin restores the score to 3, improving enamel surface quality after 14 days and restoring it back to the healthy structure (score of 2) after 21 days.

Anti-Kallikrein ensures a score of 5 after 14 days of treatment, indicating good re-mineralization, which continues with the surface improvement up to 21 days, resulting in a score of 3. The DIAGNOdent shows that Anti-Amelogenin is more effective for the remineralization of the enamel surface than Anti-Kallikrein.

### 2.2. Micro- and Nanostructure Assessment

**Figure 2:**
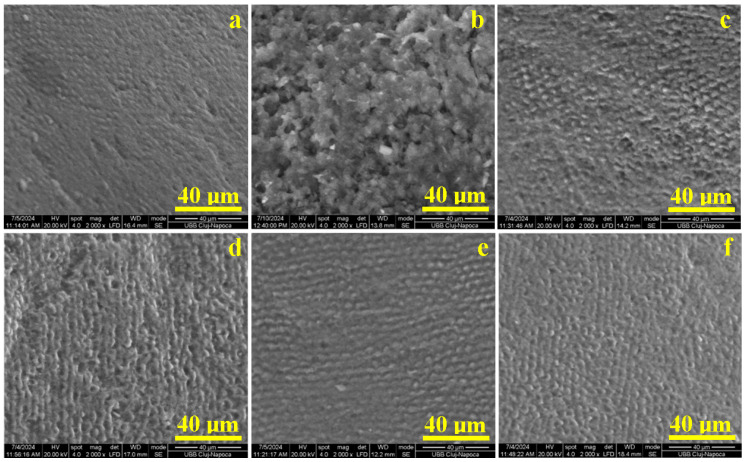
SEM images of the general aspect of the enamel surface microstructure for (**a**) healthy—untreated, (**b**) demineralized, (**c**) treated with S1 for 14 days, (**d**) treated with S2 for 14 days, (**e**) treated with S1 for 21 days, and (**f**) treated with S2 for 21 days.

The healthy, untreated enamel reveals a smooth and uniform surface, with the tops of the hydroxyapatite prisms appearing as small circles 5 µm in diameter and well embedded in a dense microstructure (Figure 2a).

The acid demineralization dissolves the hydroxyapatite nanostructural clusters, carving deeper depressions on the tops of the prisms, enhancing the prismatic interface and generating an eroded pattern with a honeycomb resemblance (Figure 2b). There is a significant mineral loss from the enamel surface, in good agreement with the data found in the literature [23,24].

The first 14 days of treatment induce advanced remineralization of the acid-etched enamel by using S1 gel, which restores the prisms’ consistency by adding new hydroxyapatite layers that progressively cover the microstructural depressions (Figure 2c). Large areas where the prisms are completely remineralized can be observed. A significant remineralization is induced by S2 after 14 days of treatment (Figure 2d) on the honeycomb margins, which become thicker, and the depression depth is significantly reduced but not completely filled.

The general aspect of the enamel microstructure is completely restored after 21 days of the treatment with both S1 and S2 gels (Figure 2e,f). The honeycomb structure is completely filled with the newly added mineral. In both cases, the remineralized surface is comparable with the healthy enamel in Figure 2a.

**Figure 3:**
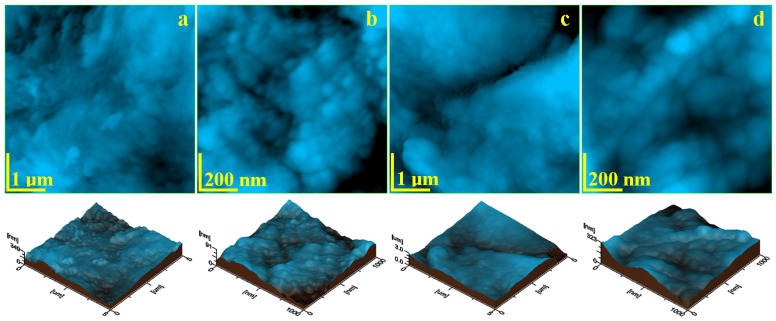
AFM topographic images of the control samples: positive control sample—healthy untreated enamel: (**a**) fine microstructure, (**b**) nanostructure, and negative control sample—demineralized enamel: (**c**) fine microstructure, (**d**) nanostructure. The tridimensional profile is presented below each topographic image.

The enamel’s fine microstructure is situated on the surface of a single hydroxyapatite prism, which implies a scanning area of 5 µm × 5 µm for the AFM investigation. Healthy, untreated enamel has a relatively uniform topography (Figure 3a) based on the dense formation of nanostructural units strongly bound to each other, with only some superficial irregularities observed. These irregularities are formed by the natural wear of the tooth surface under masticatory forces; tough food particles might exert high abrasion, which generates minor excoriations of most of the outer layers of the enamel while leaving the bulk layers untouched [25,26]. The cohesion within the enamel’s structural units is confirmed by its nanotopography (Figure 3b), where rounded clusters 40 nm in diameter are observed. They are closely packed in a dense and uniform mass, which ensures a smooth topography at the nanostructural, level resulting in an Ra of 9.78 nm and an Rq of 12.7 nm. The roughness at the nanostructural level of the enamel is one of the most important quality indicators. The literature shows common roughness values of Ra of about 10 nm and of Rq of about 12.5 nm [27,28], confirming the good quality of the positive control sample.

Acid demineralization dissolves the hydroxyapatite nanoparticles, inducing the destabilization of the nanostructural clusters, which leads to a change in their consistency and a diameter increase due to the loss of the connectivity with the protein binder. This further leads to a significant mineral loss, which forms the honeycomb structure observed by SEM microscopy [29,30]. Thus, the fine microstructure of the demineralized enamel reveals an irregular topography (Figure 3c). The mineral loss erodes the inside area of the HAP prism, generating the topographic depression (left lower side of Figure 3c), while its margins become a local crest crossed by deep erosion ditches (right upper corner of Figure 3c), which certainly increases the surface roughness.

The destructive effect of acid demineralization is better observed at the nanostructural level in Figure 3d. The nanoclusters are deeply destabilized, with their diameter increasing to about 80 nm, indicating a significant weakening of the cohesion with the protein binder. This should be related to a significant decrease in the enamel microhardness. However, the swelling of the nanoclusters and local mineral loss causes a severe increase in the nanoroughness at Ra = 27.8 nm and Rq = 36.3 nm. The negative control sample reveals all unwanted micro- and nanofeatures of the enamel surface, which must be addressed by treatment with our experimental gels.

**Figure 4:**
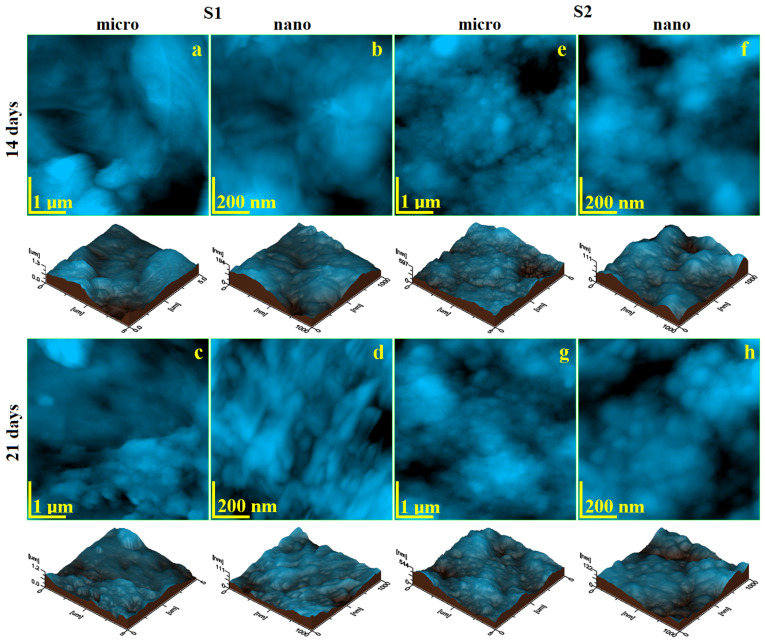
AFM topographic images of the treated enamel surface: S1 for 14 days: (**a**) fine microstructure, (**b**) nanostructure; S1 for 21 days: (**c**) fine microstructure, (**d**) nano-structure; S2 for 14 days: (**e**) fine microstructure, (**f**) nano-structure; S2 for 21 days: (**g**) fine microstructure, (**h**) nano-structure. The tridimensional profile is presented below each topographic image.

Our previous study shows that S1 gel reticulates on solid surfaces, ensuring a uniform distribution of the nano-filler and peptidic content [31]. The same reticulation is observed on the enamel’s fine microstructure after 14 days of treatment (Figure 4a). Several gel frontlines are observed to be covering the demineralized structures, facilitating the active ingredients to work directly on the enamel’s structural units. Therefore, the nanotopography in Figure 4b reveals the newly added HAP nanoparticles with elongated shapes that are strongly bound on the demineralized structures. The peptides facilitate the connection between the newly added mineral with the remains of the protein binder within the enamel surface, ensuring proper restoration. This should be related to a significant increase in the enamel microhardness. In consequence, the surface roughness decreases significantly.

The restorative effect of S1 gel is progressively enhanced after 21 days of treatment. The gel frontlines disappear from the fine microstructure’s topography (Figure 4c) due to the solid deposits of the newly added mineral over the demineralized structures, resulting in their complete restoration. Thus, the topographical aspect of the sample treated for 21 days with S1 has a strong resemblance to the healthy enamel. The nano-topography reveals a completely restored enamel, where the newly added HAP nanoparticles with a diameter of 40 nm and a length ranging between 120 and 200 nm are perfectly welded to the natural features of the enamel, ensuring the success of the remineralization process. This is sustained by the roughness value, which is comparable with the healthy enamel and with its microhardness.

**Figure 5:**
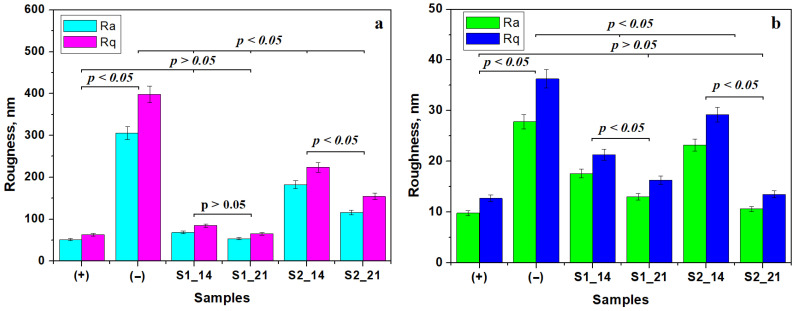
Roughness variation at (**a**) the fine microstructure level and (**b**) the nanostructure level. Error bars represent standard deviation.

Gel S2 has a finer distribution of the mineral filler that is coupled with the specific peptidic compound [31]. It produces a finer reticulation layer over the demineralized fine enamel microstructure (Figure 4e). It partly fills the demineralized gaps with newly added mineral and smoothly covers the prominent demineralization remains after 14 days of treatment. This is a significant achievement that reduces the surface roughness and should increase the microhardness. The re-mineralization process is more evident at the nano level (Figure 4f), where the finest mineral intake is progressively attached to the demineralized features through the peptide action, restoring the connection with the weakened protein binder. The situation is considerably improved by increasing the treatment period to 21 days. The fine microstructure is completely restored (Figure 4g) and has a strong resemblance to the healthy enamel, which can be seen by looking at the roughness variation in Figure 5a. The enamel’s nanotopography after 21 days reveals the restoration of nanoclusters with a diameter of about 50 nm, which are welded well into a compact and smooth structure, which ensures a roughness very close to that of healthy enamel.

The variation in roughness at the fine microstructure level reveals a significant statistical difference between the positive and negative control samples [32]. The positive sample has low roughness and the negative sample has the highest roughness. The negative sample forms a statistically relevant group. The positive sample forms a statistically significant group with the samples treated with S1 for 14 and 21 days. This indicates that the enamel roughness at the fine microstructure level is already restored after 14 days of treatment and further consolidated up to 21 days. Statistical differences were observed between samples treated with S2 at 14 and 21 days. The observed roughness decrease shows that extending the treatment period to 21 days ensures a proper consolidation of the remineralization layer [33]. Unfortunately, the fine microstructure roughness after complete consolidation with S2 has a statistical mismatch with the healthy enamel and a strong difference to the negative control. Thus, it can be concluded that the S2 samples form another relevant statistical group, which shows the success of the remineralization.

The roughness variation at the nano level is presented in Figure 5b. It reveals the simplest situation, with a significant statistical difference between the positive and negative control samples. Statistical differences between the roughness values after 14 days are observed, which significantly decrease after 21 days of treatment for both S1 and S2. Finally, the roughness values after 21 days of treatment with both S1 and S2 form a statistically relevant group with the positive control. This confirms the complete restoration of the enamel’s nanostructure achieved by both experimental gels.

### 2.3. Microhardness

**Figure 6:**
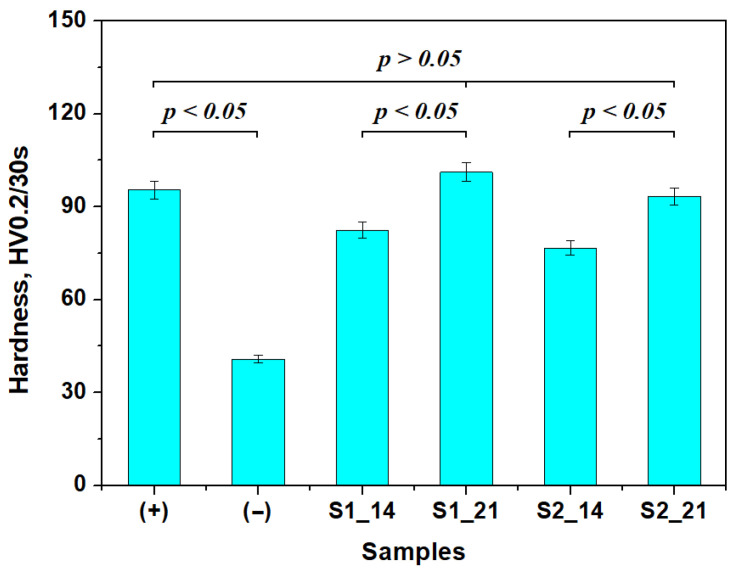
Mean hardness variation with the applied treatments. Error bars represent standard deviation.

The positive control (e.g., healthy untreated enamel) has a mean Vickers hardness of 95.49 HV0.2, which considerably decreases to 40.75 HV0.2 as consequence of the acid demineralization (Figure 6). The mineral loss affects the protein binder of the enamel, diminishing its cohesion; thus, the Vickers indenter punches a much softer material than usual. This represents a significant statistical difference between the positive and negative control samples.

Hydrogels are widely used as scaffolds in tissue engineering, as they maintain well-defined 3D structures that offer mechanical support to cells and replicate the natural extracellular matrix (ECM), enabling the transfer of nutrients and cytokines. Consequently, recent research on enamel regeneration emphasizes biomimetic mineralization of enamel without involving cells [18].

Enamel remineralization, or amelogenesis, takes place in a tightly regulated environment governed by organic substances. Interactions between proteins, protein–mineral complexes, and protein–protease mechanisms promote the growth and formation of needle-shaped hydroxyapatite crystals [20,21]. The prism-like arrangement of these crystals within the enamel plays a vital role in enhancing the tissue’s mechanical properties, offering protection against cariogenic bacteria and resistance to mechanical forces during mastication.

The proactive action of the peptide within S1 sample activates the protein binder within the enamel surface, facilitating fixation of the newly added hydroxyapatite particles and restoring the eroded areas. In consequence, the surface hardness increases to about 82.47 HV0.2 after the first 14 days of treatment, representing a good achievement. Continuing the treatment up to 21 days, the hardness becomes 101.18 HV0.2. This is a mean value comparable to the positive control sample. The statistical analysis reveals a significant difference in hardness at 14 and 21 days.

The peptide within the S2 sample also has a proactive effect on the demineralized protein binder, restoring the enamel’s micro- and nanostructural features. Therefore, the hardness increases to 76.71 HV0.2 after the first 14 days of treatment and further increases to 93.37 HV0.2 after 21 days, a fact sustained by the statistical analysis.

Figure 6 reveals that the positive sample and samples S1 and S2 after 21 days of treatment form a statistically relevant group, proving the success of the remineralization.

The DIAGNOdent results show moderate wear of the healthy enamel as a consequence of chewing. Its acidic attack has led to major indentations and enamel deterioration, a fact highlighted by DIAGNOdent. The score obtained is consistent with the altered microstructure revealed by SEM, as well as the increased roughness highlighted by AFM.

The reduction in HAP crystals from the surface leads to leveling of the micro- and nanostructural units, as evidenced by the Vickers microhardness (in agreement with the literature data) [16,17].

Amelotin (AMTN), an enamel-related protein, has been demonstrated to promote mineralization both in vitro and in vivo [34,35,36,37,38]. The rate at which minerals form is influenced by factors such as supersaturation, temperature, and the organic matrix. For instance, organic matrices, especially proteins, can accelerate the nucleation process and support the growth of crystals [9,18,22]. Therefore, the effect of Anti-Amelogenin peptides combined with the presence of saliva and nanohydroxyapatite in the tested hydrogels enhances the protein units in the demineralized enamel, ensuring the adhesion of HAP crystals adsorbed on the surface and their incorporation into the enamel structure. This fact is demonstrated by AFM images, which show the nano-HAP particles’ adhesion. Therefore, we observe that the enamel treated with hydrogel containing Anti-Amelogenin leads to the formation of a more uniform and compact remineralized surface, as proven by the roughness and microhardness being close to that of dental enamel [23,26,37].

The Anti-Kalikerin peptide also presents a good effect in regenerating dental enamel, but it has a weaker potentiation at the level of the protein units in the damaged dental enamel, which leads to a good regeneration of the enamel, though not as efficient as in the case of Anti-Amelogenin. This aspect is confirmed by the variation in the surface roughness and microhardness. The obtained results clearly evidence the benefit of Anti-Amelogenin and Anti-Kallikrein for enamel remineralization, rejecting the first part of the null hypothesis. Anti-Amelogenin has better results in restoring the surface roughness and ensuring a proper hardness compared to Anti-Kallikrein, which has a slightly weaker effect. Therefore, the second part of the null hypothesis is also rejected.

## 3. Conclusions

An analysis of enamel surfaces using DIAGNOdent, SEM, and AFM is performed before and after demineralization and after remineralization with the two peptide-based hydrogels at 14 and 21 days.

The DIAGNOdent scores obtained from the evaluation of enamel surfaces indicate that after 21 days of treatment with S1 (Anti-Amelogenin) and S2 (Anti-Kallikrein) hydro-gels, the remineralized surface is comparable to that of healthy enamel. The remineralization process is attributed to peptides, which facilitate the bonding of newly added minerals to the residual protein binder on the enamel surface. AFM images confirm the adhesion of HAP nanoparticles.

Treatment with the Amelogenin hydrogel results in a more uniform and compact remineralized surface, with roughness and microhardness values close to those of natural tooth enamel.

The Anti-Amelogenin peptide is more effective regarding remineralization, achieving better microhardness afterwards than Anti-Kallikrein after 14 days of treatment and completely restoring the enamel microhardness after 21 days. Anti-Kallikrein restores the hardness of 90% of the healthy enamel.

While the Anti-Kallikrein peptide also shows a positive effect on enamel regenera-tion, its interaction with protein units in damaged enamel is less effective, making it less efficient compared to Anti-Amelogenin.

Anti-Amelogenin is more effective regarding remineralization compactness, achieving a better microhardness than the Anti-Kallikrein peptide after 14 days of treatment and completely restoring the enamel microhardness after 21 days. Anti-Kallikrein restores the hardness of about 90% of the healthy enamel.

## 4. Materials and Methods

### 4.1. Materials

For these investigations, two experimental hydrogels were used: Anti-Amelogenin, X isoform, ABT260 (S1) (EMD Millipore Corp, USA Affiliate of Merck KGaA, Darmstadt, Germany), and Anti-Kallikrein L1, K3014 (S2) (Sigma-Aldrich, Inc., Saint Louis, MO, USA). These hydrogels were characterized in a previously published paper [31].

### 4.2. Protocol of Demineralization/Remineralization of Enamel Surface

This in vitro study was approved by the Ethics Committee of the University of Medicine and Pharmacy “I. Hatieganu” Cluj-Napoca (No. 157/10.06.2022).

Patients’ informed consent for orthodontic treatment and the use of extracted teeth for scientific purposes were obtained prior to the beginning of the study.

Sixty premolars extracted for orthodontic purposes, free of caries, stains, fissures, cracks, irregularities, abnormalities, hypoplasia, or fillings, and found to be in standard condition upon inspection, were included in this in vitro study.

After extraction, the soft tissue and calculus were manually removed using hand scalers, and the organic debris was cleaned with pumice. The teeth were stored at 37 °C in artificial saliva, with a pH of 7.4, which contained 1.5 mmol/L CaCl_2_ (Chempur, Piekary Slaskie, Poland), 50 mmol/L KCl (Chempur, Piekary Slaskie, Poland), and 20 mmol/L tris—tris (hydroxymethyl) aminomethane (Merck, Damstadt, Germany) until use.

**Figure 7:**
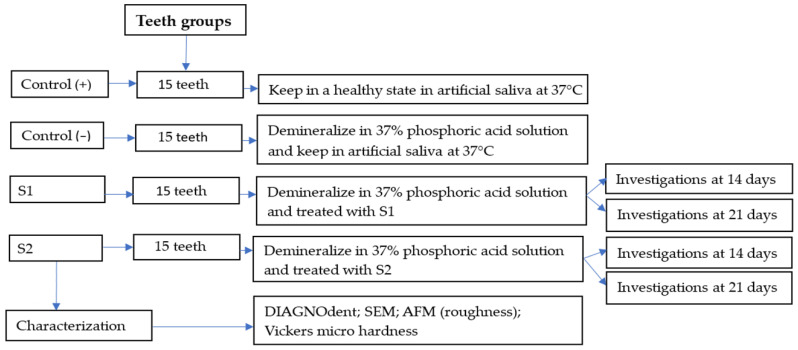
Sample size distribution scheme.

Sixty extracted teeth, intended for orthodontic purposes and free from caries and lesions, were divided into three groups of 15 teeth each (Figure 7).

The positive control (+) contained 15 healthy teeth preserved in their initial condition. The other teeth were demineralized by immersion in a 37% phosphoric acid solution for 20 min. After immersion, the teeth were rinsed with distilled water and placed in artificial saliva at 37 °C in a thermostatic water bath, with no additional treatments applied. Fifteen of the demineralized teeth were kept as the negative control (−). Another 15 were treated with S1 and 15 were treated with S2. Five teeth were sent for analyses after 14 days of treatment, and the other were treated until 21 days and afterwards subjected to the analyses.

The hydrogel was applied to the dental surface with a microbrush (a layer of 1.5–2 mm). The gel was left to act for 3 min, then rinsed with distilled water, and the tooth was placed in artificial saliva at 37 °C in a thermostatic water bath.

### 4.3. Characterizations

#### 4.3.1. Measurement of Samples with DIAGNOdent

Evaluation of the enamel surface after demineralization and remineralization at 14 and 21 days with experimental hydrogels was performed with a DIAGNOdent pen (KaVo Dental Austria GmbH, Wien, Austria). The working parameters were laser power 1 mN, laser beam wavelength 650 nm, and radiant intensity up to 130 mW/sr.

Before each use on a patient, the DIAGNOdent device was calibrated using the provided ceramic standard. A zero baseline reading was then obtained by measuring the fluorescence from a healthy area on the labial surface of the teeth. The device was recalibrated every ten readings using the ceramic standard. The DIAGNOdent tip was aimed at the enamel surface and rotated along a vertical axis. Three readings were taken, and the highest value was recorded. DIAGNOdent readings, ranging from 0 to 99, were classified into four scores based on a prior classification system, which correlates the readings with lesion progression [17,18]. Score 1: 0–4 (healthy tooth structure), score 2: 5–10 (outer half enamel caries), score 3: 11–20 (inner half enamel caries), and score 4: 21+ (dentin caries). Lesions were assessed at baseline and after 14 and 21 days.

#### 4.3.2. The Scanning Electron Microscopy (SEM)

Scanning electron microscopy (SEM) investigation was conducted using an Inspect S50 SEM Microscope produced by FEI Company, Hillsboro, OR, USA, operated in the low vacuum mode at an acceleration voltage of 30 kV.

#### 4.3.3. Atomic Force Microscopy (AFM)

Atomic force microscopy (AFM) was executed with a Jeol Scanning Probe Microscope JSPM 4210 produced by Jeol Company, Tokyo, Japan. NSC 15 hard cantilevers with a resonant frequency of 325 kHz and a force constant of 40 N/m were employed, operating in tapping mode at a scanning rate of 1 to 3 Hz, depending on the scan size. The finer microstructural features of the enamel surface were observed at a scan size of 5 µm × 5 µm, while the enamel nanostructure was examined at a scan size of 1 µm × 1 µm. Relevant images were obtained from at least three different macroscopic sites on the sample surface. The surface roughness was measured for each scanned area using Ra and Rq parameters. The mean roughness values were calculated and analyzed statistically.

#### 4.3.4. The Hardness of the Enamel Surface

Hardness tests were performed using the Duramin-40 AC3 apparatus (Struers GmbH, Fellbach, Germany). The Vickers test was conducted with a 200 gf load, 10× magnification, and an indentation time of 15 s. For each sample, 10 measurements were taken, and the mean of these results was calculated.

#### 4.3.5. Statistical Analysis

Roughness and micro-hardness mean values were calculated on five determinations performed on five similar specimens. The statistical analysis was effectuated with Microcal Origin 2019 (9.6) software, Microcal Company, Northampton, MA, USA. An ANOVA test was carried out, followed by Tukey’s post hoc test at a significance level of *p* = 0.05.

## Data Availability

The original contributions presented in the study are included in the article; further inquiries can be directed to the corresponding author.
